# Supplementary material for: Control of parallel hippocampal output pathways by amygdalar long-range inhibition
Source: eLife. 2021 Nov 30;10:e74758. doi: 10.7554/eLife.74758 (PMC8654375; doi:10.7554/eLife.74758)
Supplement: Supplementary file 1. [file elife-74758-supp1.docx]

**STATISTICAL SUMMARY**

| **Figure** | **Descriptors** | **n** | **Test used** | **Statistic** | **p-value** |
| --- | --- | --- | --- | --- | --- |
| 2c | Synapsin -70 mV  Amplitude (pA)  +/- NBQX | 9 neurons  from 8 mice | Paired t-test  *(log transformed data)* | t_(8)_ = 10.04 | 0.000008 |
| 2e | Synapsin 0 mV  Amplitude (pA)  +/- GZ | 12 neurons  from 10 mice | Paired t-test  *(log transformed data)* | t_(11)_ = 11.72 | 1.48 x10^-7^ |
| 2g | CaMKii 0 mV  Amplitude (pA)  Baseline  +NBQX  +GZ | 3 neurons  from 2 mice | Repeated-measures ANOVA  *(log transformed data)*  Tukey post hoc test  *Baseline vs NBQX*  *Baseline vs GZ*  *NBQX vs GZ* | F_(2,4)_ = 23.4  t_(2)_ = 4.73  t_(2)_ = 4.84  t_(2)_ = 0.12 | 0.006  0.001  0.001  0.9 |
| 2i | vGAT 0 mV  Amplitude (pA)  Baseline  +NBQX  +GZ | 6 neurons  from 6 mice | Repeated-measures ANOVA  *(log transformed data)*  Tukey post hoc test  *Baseline vs NBQX*  *Baseline vs GZ*  *NBQX vs GZ* | F_(2,10)_ = 10.03  t_(2)_ = 0.05  t_(2)_ = 4.12  t_(2)_ = 4.16 | 0.004  0.9  0.001  0.001 |
|  |  |  |  |  |  |
| 3h | Resting Potential (mV)  BA, NAc, PFC | BA = 9 neurons  NAc = 7 neurons  PFC = 7 neurons  from 5 mice | One-Way ANOVA | F_(2,19)_ = 1.38 | 0.27 |
| 3h | Inpit Resistance (Ohms)  BA, NAc, PFC | BA = 9 neurons  NAc = 7 neurons  PFC = 7 neurons  from 5 mice | One-Way ANOVA | F_(2,19)_ = 1.77 | 0.20 |
| 3h | Sag Amplitude (mV)  BA, NAc, PFC | BA = 9 neurons  NAc = 7 neurons  PFC = 7 neurons  from 5 mice | One-Way ANOVA | F_(2,19)_ = 1.40 | 0.27 |
|  |  |  |  |  |  |
| 4c | BA:NAc -70 mV  Amplitude (pA) | 9 pairs of neurons  from 5 mice | Wilcoxon Rank Sum | W = 15 | 0.43 |
| 4f | BA:PFC -70 mV  Amplitude (pA) | 8 pairs of neurons  from 7 mice | Wilcoxon Rank Sum | W = 0 | 0.0018 |
| 4i | BA:NAc vGAT 0 mV  Amplitude (pA) | 7 pairs of neurons  from 5 male mice | Wilcoxon Rank Sum | W = 0 | 0.016 |
| 4l | BA:PFC vGAT 0 mV  Amplitude (pA) | 7 pairs of neurons  from 1 male, 3  female mice | Wilcoxon Rank Sum | W = 0 | 0.016 |
|  |  |  |  |  |  |
| 5k | BA:NAc local vGAT 0 mV  Amplitude (pA) | 10 pairs of neurons  from 3 mice | Wilcoxon Rank Sum | W = 2 | 0.006 |
| 5n | BA:NAc CaMKii -70 mV  Amplitude (pA) | 10 pairs of neurons  from 5 mice | Wilcoxon Rank Sum | W = 22 | 0.625 |
| 5p | BA:NAc CaMKii 0 mV  Amplitude (pA) | 10 pairs of neurons  from 5 mice | Wilcoxon Rank Sum | W = 3 | 0.04 |
|  |  |  |  |  |  |
| 7c | GFP vs ChR2  p stimulated side | GFP = 6 mice  ChR2 = 8 mice | t-test | t_(5.9)_ = 2.61 | 0.041 |
| 7c | GFP vs ChR2  distance travelled | GFP = 6 mice  ChR2 = 8 mice | t-test | t_(9.2)_ = 1.27 | 0.23 |
| 7f | Voltage change in  SalB (mV) | 7 neurons  from 3 mice | Repeated measures ANOVA  Effect of time | F_(24,144)_ = 5.94 | 2.64 x10^-12^ |
| 7i-k | DMSO vs SalB  Control vs KORD | Cont = 9 mice  KRD = 7 mice | Mixed ANOVA  Effect of group  Effect of drug  Interaction | F_(1,14)_ = 15.97  F_(1,14)_ = 15.06  F_(1,14)_ = 7.45 | 0.001  0.002  0.016 |
| 7i | DMSO vs SalB  p stimulated side | 9 mice | Paired t-test | t_(8)_ = 1.1 | 0.30 |
| 7i | DMSO vs SalB  distance travelled | 9 mice | Paired t-test | t_(8)_ = 0.91 | 0.39 |
| 7k | DMSO vs SalB  p stimulated side | 7 mice | Paired t-test | t_(6)_ = 4.62 | 0.004 |
| 7k | DMSO vs SalB  distance travelled | 7 mice | Paired t-test | t_(6)_ = 1.21 | 0.27 |
|  |  |  |  |  |  |
| 8c | GFP vs ChR2  p stimulated side | GFP = 4 mice  ChR2 = 7 mice | t-test | t_(6.4)_ = 0.40 | 0.70 |
| 8c | GFP vs ChR2  distance travelled | GFP = 4 mice  ChR2 = 8 mice | t-test | t_(6.9)_ = 0.08 | 0.94 |
| 8f | Voltage change in  SalB (mV) | 3 neurons  from 2 mice | Repeated measures ANOVA  Effect of time | F_(19,38)_ = 2.95 | 0.002 |
| 8i-k | DMSO vs SalB  Control vs KORD | Cont = 9 mice  KRD = 7 mice | Mixed ANOVA  Effect of group  Effect of drug  Interaction | F_(1,14)_ = 3.56  F_(1,14)_ = 3.00  F_(1,14)_ = 10.85 | 0.08  0.11  0.005 |
| 8i | DMSO vs SalB  p stimulated side | 9 mice | Paired t-test | t_(8)_ = 1.01 | 0.34 |
| 8i | DMSO vs SalB  distance travelled | 9 mice | Paired t-test | t_(8)_ = 0.66 | 0.52 |
| 8k | DMSO vs SalB  p stimulated side | 7 mice | Paired t-test | t_(6)_ = 3.14 | 0.02 |
| 8k | DMSO vs SalB  distance travelled | 7 mice | Paired t-test | t_(6)_ = 4.15 | 0.006 |
|  |  |  |  |  |  |
