# Supplementary material for: Control of parallel hippocampal output pathways by amygdalar long-range inhibition
Source: eLife. 2021 Nov 30;10:e74758. doi: 10.7554/eLife.74758 (PMC8654375; doi:10.7554/eLife.74758)
Supplement: Supplementary file 2. [file elife-74758-supp2.docx]

**SUPPLEMENTARY STATSTICS SUMMARY**

| **Figure** | **Descriptors** | **n** | **Test used** | **Statistic** | **p-value** |
| --- | --- | --- | --- | --- | --- |
| S2 - 1f | With direct inhibition  Amplitude (pA)  Baseline  +NBQX  +GZ | 4 | Repeated-measures ANOVA  *(log transformed data)*  Tukey post hoc test  *Baseline vs NBQX*  *Baseline vs GZ*  *NBQX vs GZ* | F_(2,6)_ = 7.9  t_(3)_ = 0.7  t_(3)_ = 4.7  t_(3)_ = 3.5 | 0.02  0.7  0.001  0.002 |
| S2 - 1f | No direct inhibition  Amplitude (pA)  Baseline  +NBQX  +GZ | 8 | Repeated-measures ANOVA  *(log transformed data)*  Tukey post hoc test  *Baseline vs NBQX*  *Baseline vs GZ*  *NBQX vs GZ* | F_(2,14)_ = 84.7  t_(7)_ = 12.1  t_(7)_ = 12.7  t_(7)_ = 0.53 | 1.5 x10^-8^  0.001  0.001  0.84 |
|  |  |  |  |  |  |
| S7 - 1b | Pre vs post SalB | vH-BA = 3  vH-NAc = 7 | Mixed ANOVA  Effect of group  Effect of drug  Interaction | F_(1,8)_ = 0.29  F_(1,8)_ = 13.8  F_(1,8)_ = 0.35 | 0.6  0.007  0.57 |
| S7 - 1c | Pre vs post SalB | vH-BA = 2  vH-NAc = 6 | Mixed ANOVA  Effect of group  Effect of drug  Interaction | F_(1,6)_ = 0.21  F_(1,6)_ = 31.3  F_(1,6)_ = 0.75 | 0.66  0.001  0.42 |
|  |  |  |  |  |  |
